# What contributes to the long-term implementation of an evidence-based early childhood intervention: a qualitative study from Germany

**DOI:** 10.3389/frhs.2023.1159976

**Published:** 2024-01-19

**Authors:** Marie Lisanne Schepan, Tanja Jungmann, Sören Kliem, Carolin Siegert, Malte Sandner, Tilman Brand

**Affiliations:** ^1^ Department of Prevention and Evaluation, Leibniz Institute for Prevention Research and Epidemiology—BIPS, Bremen, Germany; ^2^ Department of Public Health, University of Bremen, Bremen, Germany; ^3^ Department of Special Needs Education and Rehabilitation, Carl von Ossietzky University of Oldenburg, Oldenburg, Germany; ^4^ Department of Social Work, Ernst-Abbe-Hochschule Jena, University of Applied Sciences, Jena, Germany; ^5^ Department of Business Administration, Nuremberg Institute of Technology, Nuremberg, Germany

**Keywords:** sustainability, evidence-based interventions, implementation, early childhood interventions, qualitative research

## Abstract

**Background:**

Rigorous research trials have demonstrated that early childhood interventions can reach socially disadvantaged families and can have a lasting impact on the healthy development of their children. However, little is known about the internal and contextual factors that contribute to the long-term implementation of such interventions. In this study, we investigated the development of the home visiting program Pro Kind. The program was adapted from the evidence-based US-American Nurse-Family Partnership program and was implemented in Germany in 2006. Using an exploratory approach, we examined factors contributing to the long-term implementation of this program.

**Methods:**

Qualitative interviews with program implementers (midwives, social workers, program managers) of the Pro Kind program and key stakeholders in two cities in Germany were conducted. Interview guides were developed to assess participants' perceptions and experiences on how the program had developed over time internally and in the interaction with its environment. Data were collected between March and September 2021. Drawing on the Consolidated Framework for Implementation Research (CFIR), data was coded according to the principles of thematic analysis.

**Results:**

A total of 25 individuals (11 program implementers, 14 key stakeholders) were interviewed. The identified factors related to three out of five domains of the CFIR model in our analysis. First, regarding the *intervention characteristics*, the evidence of effectiveness and the relative advantage of the implementation of the program compared to similar interventions were viewed as contributors to long-term implementation. However, the program's adaptability was discussed as a constraining factor for reaching the target group. Second, concerning the *inner setting*, stakeholders and program implementers perceived the implementation climate, the leadership engagement and the program's size as relevant factors for networking strategies and program visibility. Third, as part of the *outer setting*, the degree of networking with external stakeholders was highlighted of great importance for the program.

**Conclusions:**

We identified several factors of particular importance for the long-term implementation and sustainability of an early childhood intervention at the practice level, particularly in the local context in Germany. These findings should inform the design of impactful, scalable, and sustainable early childhood interventions targeting disadvantaged families.

## 1 Introduction

Children exposed to early childhood adversities, such as poor socioeconomic conditions, maltreatment and neglect or unhealthy family functioning, are at increased risk for poor physical and mental health and low educational success (1–3). A promising approach to reach and support socially disadvantaged families is home-based interventions in early childhood. Research trials show lasting effects of these interventions on mother and child health outcomes (4–8). However, less is known about the factors that contribute to the “survival” of such interventions in real-world settings after initial project funding has ended (9, 10). Discrepancies between research settings and the context in which the interventions are implemented are a fundamental challenge for sustaining evidence-based public health interventions. Furthermore, over time interventions evolve due to factors such as changing populations, policies, available resources, and organizational structures, which may have positive (refinement of program delivery) or negative implications (loss of fidelity, discontinuation of program) (11). The identification, description, and understanding of internal and external factors, as well as how they interact to influence long-term implementation, is hence essential to maintain program continuation and effectiveness, further optimize the intervention benefits, and prolong program sustainability (6). By long-term implementation we mean the continuation of a public health program after the initial, project-based implementation that was supported by external (research) funding. Long-term implementation has also been described as program sustainability (10). Research on implementation processes and sustainability is needed to plan proactively for program continuation and to support programs in unfolding their full potential (12, 13).

There is a wide range of terminologies for relevant constructs, and an abundance of frameworks and models identifying factors that are important for the implementation process of health interventions (14, 15). Regarding the evaluation of early childhood interventions, previous research has revealed that contextual factors, as well as the dynamic interplay between the program and its environment, play a crucial role (16, 17). Previous studies mostly investigated earlier stages of implementation, focusing on constructs such as fidelity, dosage and quality of early childhood interventions (18, 19). However, for a comprehensive evaluation of the success of early childhood interventions, it is essential to understand the adoption, scale-up, and sustainability of interventions that have been in place within communities for some years (20). To date, only a few studies have investigated factors that are related specifically to long-term implementation of early childhood interventions, focusing mainly on settings in the US (21–24). The factors identified in these studies include the consideration of the powerful role of context (e.g., community characteristics, addressing service context) as well as the impact of other factors such as program delivery (e.g., service dosage, staffing, program flexibility) (21, 22). For instance, in the Nurse-Family Partnership program (NFP), a large home visiting program from the US, analyses of implementation and outcome data helped the identification of issues specific to certain contexts (23). The results of a mixed method analysis of participant attrition showed, for example, that home visitors in high retention sites adapted the program more completely to their clients' needs and used less directive and prescriptive approaches. Hence, a flexibilisation of the program led to adaptations of the program guidelines, nurse education, visit frequency, content, and location of visits (24).

In this study, we investigate the long-term implementation of the prenatal and infancy home visiting program Pro Kind (25, 26). The program is based on the NFP program (27) and was adapted to the German context. The aims of the program, which focuses on psychosocially and economically disadvantaged families, are to enhance maternal and child health and to reduce the risk of child abuse and neglect. Professional home visitors (midwives or social workers) support first-time mothers from pregnancy to the child's second birthday. The home visits start during the second trimester of pregnancy and are generally then scheduled for every other week. The home visitors work with the families following a structured topic guide covering a wide range of issues including e.g., maternal health, healthy family routines, and life-course planning. In sum, the key features of the Pro Kind program are its tightly defined target group criteria (only first-time mothers, socially disadvantaged, start during second trimester of pregnancy), its thematically comprehensive and structured approach and its duration. These elements are essential for achieving the desired outcomes for children and families (27).

The development of the Pro Kind program is closely tied to changes that occurred at the national level at the time of its conception. A national early childhood intervention program (ECI) was initiated in 2006 (28, 29). In this context, the Pro Kind program was one of several pilot projects to receive additional funding at the federal state level. It started in 2006 with a multicenter randomized controlled trial (RCT) in 15 sites located in three federal states (Bremen, Lower Saxony, Saxony) and ended in 2012. After this phase, the program materials were revised substantially in close cooperation with the National Center for Early Prevention. The key features mentioned before were, however, kept. The overall sustainability of the Pro Kind program was low across the sites, as it was continued in two of the original sites.

Alongside the evaluation of program outcomes (26, 30–35), the implementation of the Pro Kind program was closely monitored to examine implementation differences (36) and the association of participant characteristics and process variables with program attrition (37). However, investigations on the long-term program development that assess different implementation levels are still needed, considering the different natures of the local implementation settings.

Therefore, we aim to explore factors that shape the long-term implementation of Pro Kind. The findings will enable us to illustrate and contrast factors contributing to the positive as well as negative program development and intervention performance.

## 2 Materials and methods

### 2.1 Study design

We conducted semi-structured interviews with program implementers (midwives, social workers, program managers) and key stakeholders (e.g., representatives of youth and welfare services, pediatricians). Qualitative methods were used to gain insights into participant's perspectives about the program development and its integration into local community structures over time. Ethical approval for the study was obtained from the ethics committee of the University of Bremen, Germany (reference number 2021-05). Participation in the interviews was voluntary, and all participants provided written informed consent. This study was conducted in line with the Consolidated Criteria for Reporting Qualitative Research recommendations (COREQ, Supplementary File 1) (38). The research team characteristics are presented in Supplementary File 2.

### 2.2 Selection of sites and site characteristics

The interviews were conducted at two German sites, Bremen and Brunswick. These cities were selected because they were the only sites, where the Pro Kind program was still being implemented since 2006. The city of Bremen has over 563.000 inhabitants and is located in Northern Germany. It is surrounded by the larger federal state of Lower Saxony, where the city of Brunswick, with about 248.500 inhabitants, is located.

The implementation conditions between the two sites differed already at program initiation. During the trial phase, 80 families in Bremen and 35 families in Brunswick took part in the Pro Kind program, reflecting the different sizes of the cities. At both sites, the program was delivered through established local social service organizations. However, in Brunswick the program was integrated into the structures and processes of the youth and welfare office to a greater extent than in Bremen. This affected in particular the procedures in recruiting families, laying with the youth and welfare office in Brunswick. With its three employees and a relatively small number of cases (about 10), the program in Brunswick has been scaled down over the past years, whereas in Bremen the number of cases has increased to 140.

### 2.3 Sampling

At study onset, the research team (TB and MLS) presented the study aims and procedures to the Pro Kind staff from both sites at an annual network meeting. The program managers facilitated contact between the research team and the midwives and social workers who were implementing the program. The sampling of the Pro kind staff was purposive in that we wanted to prioritize interviews with staff who had been working with the program for several years. Potential key stakeholders in the field of early childhood interventions were initially identified by the program managers, the interviewed staff and the research team. Thereafter, snowball sampling was applied to identify further stakeholders, continuing until no additional interview participant could be identified or data saturation was achieved. In an effort to counterbalance the snowball approach, we conducted internet searches to try and identify further potential interview participants that were not mentioned by the program implementers.

The interviews were conducted between March and September 2021 and the interviewer (MLS) did not know any of the interviewees prior to the study.

### 2.4 Interview guide and data collection

Using an exploratory approach, the research team discussed the key domains of program implementation with the program implementers at their annual network meeting and developed topic guides for each target group (program implementers and stakeholders). The topic guides were designed to assess interviewees' perceptions and experiences on how the program has developed over time internally and in the interaction with contextual factors (see Supplementary File 3 for the original interview guides and the English translations).

Depending on the COVID-19 regulations, the interviews were either conducted online (using the platform *GoTo* Meeting), by telephone or face-to-face. Where possible, the interviews took place face-to-face at the partner organization's workplace, in a closed room during normal operating hours. Regardless of the format, only the interviewee and the interviewer were present during the interview. Before the interview, all participants received the study information sheet and a consent form. The interviewees were interviewed once and did not receive any incentives for their participation. The interviews were conducted by the same researcher (MLS). The mean interview duration was 42 min (range 23–70 min). All interviews were conducted in German and were digitally audio-recorded and later transcribed verbatim. Samples of the transcripts were double-checked by reading the text while listening to the audio-recordings (MLS). Selected interview quotes were translated into English for this manuscript by MLS and CS, and TB cross-checked the translations (see Supplementary File 4 for the original quotes and the English translations).

### 2.5 Data analysis

Interview transcripts were coded in MAXQDA (version 2020). The analysis followed the phases of thematic analysis (39). To identify patterns in the data, we employed a hybrid inductive-deductive approach. Despite the exploratory nature of our data collection, the inductive analysis revealed certain themes and codes that increasingly aligned with a widely used implementation framework known as the Consolidated Framework for Implementation Research (CFIR). The CFIR offers a comprehensive typology categorizing barriers and facilitators associated with implementation (40). It comprises 39 constructs organized into five domains: Intervention Characteristics, Outer Setting, Inner Setting, Characteristics of Individuals, and Processes. Initially, all interviews were coded inductively by MLS. To obtain different perspectives on the coding scheme, two research assistants independently coded two interviews. Where differences occurred, MLS and the research assistants discussed the codes and the coding scheme was adapted accordingly. After the first round of coding, a second round was carried out by MLS to refine the codes. The codes were then collated and classified under the domains of the CFIR-model. An example of the coding frame used to classify codes under the CFIR-domains is provided as Supplementary File 5. In the last step, underlying themes deemed to be of central meaning for the long-term implementation of the program were identified. The results were presented to the research team and the Pro Kind program managers several times to discuss major themes and key findings.

## 3 Results

### 3.1 Sample characteristics

A total of 25 persons, one man and 24 women, aged 29–68 years, took part in the interviews. Four were from Brunswick, and 21 were from Bremen. Eleven of the interviewees were program implementers (midwives, social workers, and program managers). Their experience of working with the Pro Kind program ranged from 5 to 16 years. The remaining 14 were stakeholders with a range of professional backgrounds, including social work, pediatrics and psychology, who were working for institutions related to early childhood interventions (e.g., child and youth welfare services, counseling centers, social security office, job centers).

### 3.2 Factors relating to long-term implementation organized under the CFIR-domains

Factors related to long-term implementation were found in three of the five CFIR-domains: Intervention Characteristics, Inner Setting, and Outer Setting. The specific factors mentioned for each of the three domains are presented hereafter (in italics) using quotes from the raw interview data.

**Figure 1:**
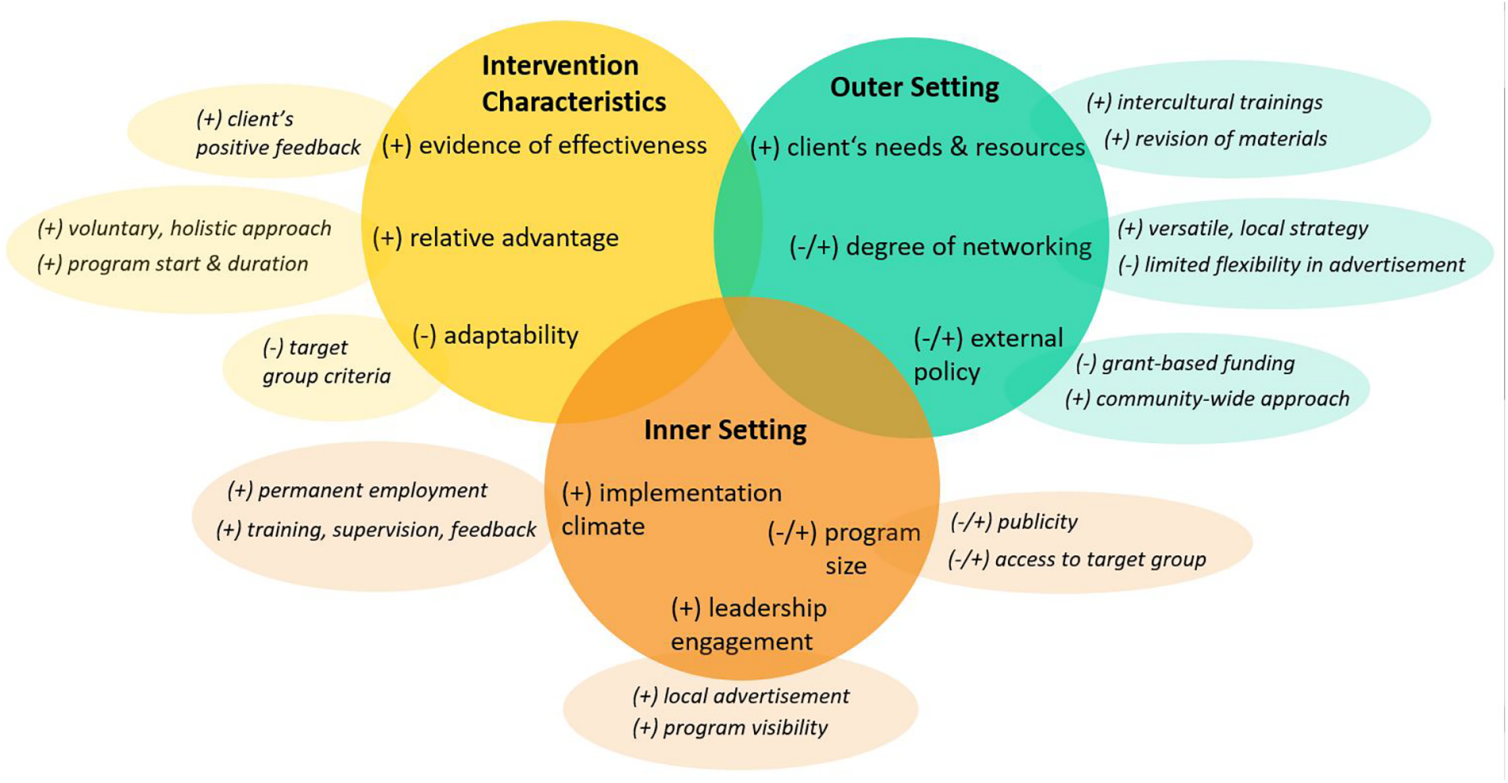
Overview of results (italics) according to the three CFIR-domains and factors relevant to our study. Notes. (+), facilitating factor; (−), hindering factor; (+/−), facilitating and hindering factor.

Figure 1 depicts an overview of the three domains, the related factors (within the big bubbles) and subfactors (within the smaller bubbles) which are each highlighted as facilitating (+) or hindering (-) factors, or both (+/-). We did not identify factors in the data that could be assigned to the CFIR-domains Characteristics of Individuals and Processes.

#### 3.2.1 Intervention characteristics

The main facilitating factors that emerged included the *evidence of the effectiveness* of the program and the *relative advantage of the implementation* of the program compared to other interventions in the field.

Based on their experience during program delivery and the client's positive feedback, most program implementers were convinced that the families benefited from program participation. One stakeholder reported on the feedback from the families as follows:

“Especially families, who at first were somehow resistant, because they could not really grasp the program at first, said afterwards that it was really good.” (stakeholder#1, Bremen)

This view was shared by many other interview participants and reinforced the stakeholders' and program implementers' general belief in the program's approach. Targeting first-time mothers, starting early during pregnancy, and providing a relatively long program duration that allows a strong working alliance (between home visitor and mother) were viewed as obvious advantages compared to other local programs in the field of early childhood interventions by program implementers and stakeholders. In addition, the holistic and voluntary approach of the program was highlighted as particularly important. However, these core components of the program lead to relatively narrow target group criteria, and this also resulted in criticism of the program's lack of *adaptability*. For example, several stakeholders regret that the program is limited to first-time mothers only, thus withholding it from other mothers with needs:

“… but they already have a clearly defined target group. And many of my clients, for example, don't fit in at all. So it’s not always first-time mothers who need this support. It is often second and third-time mothers (…).” (stakeholder#2, Bremen)

#### 3.2.2 Inner setting

Several home visitors positively emphasized aspects related to the *implementation climate* within the organization. Permanent employment contracts for midwives, which are often not offered by comparable employers, were perceived as an appreciation of the program providers and their work fostering commitment to the program. Additionally, opportunities for further training, supervision, feedback, and case consultations on a regular basis were reported to contribute to a positive learning climate. An external stakeholder commented on the working conditions as follows:

“… the impulse of the professional support and (…) the relatively conducive working conditions of the professionals, right? So professional advice, regular training, regular permanent employment of colleagues in contrast to the family midwives (…) in all other states, family midwives are not employed, but work on a fee basis, which is a disaster for this work.” (stakeholder#5, Bremen)

The implementation processes, both at the organizational level and individual level with clients, could thus be continuously reviewed and, if necessary, adjusted through the assistance and input of program providers. This ensured the sustainable quality assurance of intervention delivery, as well as the satisfaction and commitment of employees, fostering retention within the organization in the long-term.

Furthermore, *leadership engagement* emerged as a strong facilitating factor for long-term program implementation. The active engagement of program managers in advertising the program personally, their involvement in the adaptation of intervention materials (also at national level), as well as their participation in various local events of early childhood interventions were reported and highlighted by various interview participants:

“And Pro Kind is actually also active in smaller projects. So, I have already experienced that the management participated in the designing of the flyer in simple language, or I mean that they were also there when these cards for smartphone use and childcare were somehow developed, that they were also present and actively contributed.” (stakeholder#4, Bremen)

The commitment of the program managers to the program and beyond, to the promotion of early childhood interventions, was valued by stakeholders, leading to an increase in the program's visibility and fostering a trusting relationship between stakeholders and the program managers. Consequently, it promoted closer collaborations, especially regarding the referrals of families to the program.

One factor that links the inner and the outer setting is the critical role of the program's size at the two sites. It can be viewed as both, a facilitator and a barrier to long-term implementation, depending on the location. As it started with a larger team and more families, the Bremen site had a significant increase in funding and therefore in personnel. This enabled the program managers and the midwives to invest more time in networking at the city district level with the overall goal to establish collaborations with stakeholders who refer families to the program. Particularly the wide access to the target group is seen as an important prerequisite to survive in the long-term. According to stakeholders, the size of the program also played a central role in gaining publicity, to be recognized as an established partner. Responding to a question about the program's position in the local network of early childhood interventions, a stakeholder refers also to the program's size:

“I experience Pro Kind as one of the big players. So, then I immediately think, okay, big organization, many colleagues, widely known too, and very established, in my choice of words.” (stakeholder#6, Bremen)

Due to a decreasing, smaller program size at the other site, in Brunswick, this facilitating process could not be initiated yet, with a negative consequence for its visibility. In this context, the program manager reported a shortage of staff, especially local midwives, and a different funding scheme hindering the program's growth at this site. Accordingly, the program size has played a significant role in its reach, ability to act and, thus, long-term implementation.

#### 3.2.3 Outer setting

Regarding the aspect *clients' needs and resources*, the program implementers emphasized the constant social change, mainly through immigration, which resulted in ongoing diversification, also of the target group. Accordingly, working materials were revised and provided in easy-to-understand language, and program implementers were trained in intercultural competencies. Despite these efforts, one stakeholder, for example, still saw a need to expand the language diversity in the team:

“… what I experience again and again (…) is the language, the language barrier. So, in many families, the mother tongue is present, there is little German proficiency. And of course, not all midwives have these language skills. And I think we need to look again at the employees, can we also hire people who speak one language or another. (…). I think that would probably also be Pro Kind`s wish.” (stakeholder#3, Bremen)

While acknowledging room for improvement, program implementers emphasized that directing attention and adapting to clients' needs aimed to enhance the working alliance and ensured the quality of program delivery—both are considered facilitators for long-term implementation.

There was a broad agreement that the *degree of networking* with other external stakeholders was essential for getting access to the hard-to-reach families, and to provide appropriate support. The program implementers rely on the cooperation with local stakeholders in order to integrate the families into the existing community structures, such as childcare, counseling services, or activities for mothers in similar situations and to promote their self-efficacy. One program manager summarized the importance of networking as follows:

“So, networking is very important. Pro Kind without networking wouldn't work at all (…). Access is only possible through our stakeholders. And then there are specific issues. That means we see ourselves as guides for specific issues. Meaning we can tell the families that they can go there for the problem (…) and that we work together to ensure that the families manage to receive the help and support they need.” (program manager#1, Bremen)

Further, in Bremen the versatile, extensive networking through participation in workshops, expansion of local working groups, in addition to low-threshold networking through personal contact in local city districts, facilitated a general expansion of the network. This was reflected in the consistent comments of the site's stakeholders, who perceived the program as being present, well-known and established in the local network. One stakeholder noted:

“… by being present as a program not only in individual districts, but throughout Bremen, it is well known and thus also an established partner in the municipal network.” (stakeholder#4, Bremen)

However, in Brunswick, networking was perceived to be a central challenge. A fixed recruitment procedure, organized by the youth and welfare services, limited the ability of the program implementers to get in contact with other stakeholders who could refer families to the program. It also provided limited flexibility in the way of advertising the program. Home visitors were concerned that recruitment through the youth and welfare services could lead to families being wary about participating:

“It is more difficult to motivate women to join the project. Because I tell you now, in the eyes of the young mothers, who may have already had experiences with the youth and welfare office as a child, the similarity with outpatient child protection service is too large.” (home visitor#1, Brunswick)

In this context, the staff reported a stagnation and decline in the network, and thus, a decrease in the number of participating families. Besides the difficulties related to this recruitment regulation, further challenges in cooperation between the Pro Kind program and the local youth and welfare services were evident. Staff from both institutions reported difficulties in communication, lack of clarity about each other's roles and functions, and recurring tensions in the joint assessment of child protection. These difficulties were vested in the contrasting approaches and priorities of the two institutions. While both were interested in a constructive cooperation to ensure the best support for families, the Pro Kind staff placed greater value on the voluntary and preventive nature of the program. This also included a trustworthy relationship with mothers, respecting their privacy concerns. In contrast, the youth and welfare offices have a strong child protection mandate and emphasized the need for close exchange of information about critical cases.

As a part of *external policy,* the program managers, in particular, expressed uncertainty about the program's grant-based funding situation, which posed challenges to long-term planning:

“We have to re-apply every year, check again and that takes a lot of energy as well.” (program manager#2, Bremen)

One key factor repeatedly mentioned by different participants in Bremen was the integration of the Pro Kind program in a community-wide approach to foster child health in disadvantaged families. This community-wide approach combined several preventive interventions and was accompanied by a large research project. The political decision to implement a community-wide approach secured extra funding for the Pro Kind program and led to an increasing number of families that could be served. At the same time, the pressure to relax the eligibility criteria increased.

## 4 Discussion

In this study, we examined key factors related to long-term implementation of the home visiting program Pro Kind at two different sites in Germany.

Applying the CFIR-model to the analysis, we found relevant factors related to three of the five CFIR-domains: Intervention Characteristics, Inner Setting and Outer Setting. Our findings also highlight the dynamic interplay between program factors (e.g., target group criteria), organizational factors (e.g., program size) and the context of implementation (e.g., degree of networking).

Looking at the intervention characteristics, stakeholders and program implementers viewed the evidence of effectiveness and the relative advantage of the implementation of the program compared to similar interventions as contributors to long-term implementation. However, criticisms pointed to the lack of the *program's adaptability* as a constraining factor for growth, primarily because of the program's tight target group criteria. Concerning the inner setting, the implementation climate and the leadership engagement were perceived as relevant factors for staff qualification, continuity and the visibility and credibility of the program. In addition, the *program's size* emerged as an underlying factor that shaped the capacities for intensive networking, activities to increase visibility and access to the target group. Concerning the outer setting, next to the external policy and efforts to meet the clients' needs, the central importance of the *degree of networking* was highlighted. In particular, the program's relationship with the youth and welfare services emerged as challenging, mainly related to difficult access to families, tensions in communication, and different priorities.

Drawing on research on the sustainability of public health interventions, the factors and subfactors we identified from the data largely align with the three primary influences on sustainability highlighted in numerous studies: Characteristics of the intervention, factors in the organizational setting, and factors in the community environment at each intervention site. Thus, the importance of shifting the primary focus away from funding sources when designing sustainability research is highlighted (10, 41).

To some degree, there is an inherent tension between evidence of effectiveness, which relies on program integrity, and a program's adaptability and flexibility. In the field of implementation research on early childhood interventions, this challenge is well-recognized, as addressing this issue requires an understanding of theories, components, contextual influences (e.g., variation of risk exposures in families) that contribute to the effectiveness of a program (16). In the case of Pro Kind and NFP, the tightly defined eligibility criteria, the structured approach during the visits and the long program duration are hypothesized to be key elements for program effectiveness (27). Extending the target group criteria to include multiparous women has not been investigated within the RCT of the Pro Kind program, but has also been raised in other studies evaluating the NFP program (23, 42). However, this adaptation could result in reduced or no effectiveness and would entail larger changes to the program's content. Current research from the NFP is therefore investigating whether the program can be adapted to meet the higher acuity and overlapping needs of multiparous mothers (43).

Our results highlighted that a positive implementation climate, characterized by regular feedback, training, and supervision of staff, is crucial for successful implementation. This is because, as prior research shows, such a climate enhances the providers' abilities, readiness, and competencies to deliver early childhood interventions effectively (16, 17, 44, 45). Consequently, these factors influence the quality of implementation of early childhood interventions.

Our findings regarding the role of intensive networking in facilitating access to targeted families and addressing the families' needs by linking them to other resources in their communities, is in line with findings from other studies (46–48). These studies indicate that home visitors are likely to be more effective in retaining clients and in serving families with multiple needs when collaborating closely with those providing other relevant services in the local communities they serve. Moreover, continuously engaging stakeholders throughout the ongoing implementation processes might foster the fit between the intervention and the local context and the maintenance and improvement of interventions within care settings (9, 11).

Our findings also point to the critical program size, which enables or prevents program implementers to engage in intensive networking. This intensive networking is not only important for the practical work with families but also for the visibility in the stakeholder network and for political influence to sustain or increase funding. There is certainly no fixed rule for the critical size of a program and it would also be a limiting factor for a countrywide implementation if a program like Pro Kind could only be offered in larger cities to achieve an adequate size. Nevertheless, small-scale program sites may need specific strategies or extra support from other program sites for intensive networking.

As our findings confirm, the collaboration between early childhood interventions and youth and welfare services, particularly the child protection service is vital to maximize the benefits of the intervention (49). The issues reported at both sites are mostly in line with recent findings indicating the need to address misalignments of the priorities and working styles of the institutions involved (50) and the stigma associated with child protection services as well as to establish adequate communication channels between the programs to enhance collaboration and serve the same families adequately (42, 51).

### 4.1 Practical implications

From the themes that emerged from our analysis concerning the lack of adaptability, the program's size, and the degree of networking, several practical implications can be derived. These are particularly directed towards researchers and practitioners involved in program development and implementation, who must respond to continuous environmental changes to ensure the ongoing success of these programs. Adaptability is certainly a necessary trait of an intervention that tries to survive in a rapidly changing environment. As one approach for regular small-scale program adaptations, internal discussions about the appropriateness of the program materials and possibilities for further education could help an intervention remain relevant. With regard to alterations and changes that concern the whole intervention, implementation research has suggested that adding new components to an existing intervention can help to improve effectiveness (52, 53). However, changing the core components, such as the eligibility criteria, may have consequences for the appropriateness of the intervention content and the effectiveness. Ideally, such an adaptation should be accompanied by a process and outcome evaluation (54). If program implementers decide to keep the integrity of the original model, then a strong emphasis on the program's effectiveness and relative advantage over other programs may counterbalance the lack of adaptability. While contextual factors, such as external policy-making or future austerity cuts, are rather out of control for program implementers, investment in local networking seems advisable because it may be a decisive factor for maintained funding. Regarding the critical size of the program, it may be specifically important for small-scale program sites to develop strategies for effective networking. For a preventive intervention that relies on voluntary participation and a trusting working alliance with the families, it may be important to keep a critical distance to the child protection service and to be viewed as working independent from it. Nevertheless, such intervention programs need to be reliable partners for the youth and welfare services when coordinated action is necessary. Proactive role clarification and clear process descriptions for coordinated action may help to resolve this tension.

### 4.2 Limitations

The findings of this study should be interpreted considering the following limitations. Firstly, the results reported here draw upon only two sites in Germany, which may limit their generalizability. Using the CFIR-model as a theoretical framework in our analysis however helped us to present our findings on a conceptual level, thereby adding to the transferability of the findings. Secondly, although the CFIR-model is comprehensive in scope, it does not pre-specify the importance or relationship between the individual factors. Consequently, while we highlighted the factors that came through as the most relevant ones according to our analysis, we cannot claim any causal relationships between them. Due to our exploratory data collection approach, we used the CFIR-model for guiding coding, data analysis, and reporting our results. It might have been however advantageous to incorporate the CFIR-model into the data collection process earlier for capturing the factors more comprehensively. Thirdly, the number of interviews was not balanced between the two sites since we recruited only a small number of interview participants at the site where the program size decreased over time. Further insights into potential challenges of long-term program implementation from the stakeholders' perspective would have been beneficial for our analysis, but we did not identify any additional stakeholders who felt competent to discuss the program. However, considering the qualitative nature of our study, we gained fruitful information about hindering factors by including additional participants from a different context. Furthermore, following an exploratory approach, we did not collect data at the sites where the program ended after the initial study phase in 2012. This limits the generalizability of our findings. Lastly, it is possible that the snowball sampling may have resulted in a selection bias. Starting with the program implementers led us to interview stakeholders that were in close collaboration with the program. Despite additional internet searches conducted to counterbalance the snowball approach, we may have missed other stakeholders at the outskirts of the network who may have had different or more critical views on the program.

## 5 Conclusion

In this qualitative study, we identified factors of particular importance for the long-term implementation of the Pro Kind program. We highlighted issues about the program's adaptability and the critical role of intensive local networking under consideration of different program developments at two German sites. Presenting our results on a conceptual level by using the CFIR-model as a theoretical framework and giving practical implications on the program, organizational and context level may inform future adaptations, enhancements and design of early childhood interventions for socially disadvantaged families.

## Data Availability

The datasets presented in this article are not readily available because the informed consent signed by the participants did not include their agreeing to their qualitative data being shared publicly. Requests to access the datasets should be directed to Tilman Brand, brand@leibniz-bips.de.
