# Supplementary material for: What contributes to the long-term implementation of an evidence-based early childhood intervention: a qualitative study from Germany
Source: Front Health Serv. 2024 Jan 19;3:1159976. doi: 10.3389/frhs.2023.1159976 (PMC10834770; doi:10.3389/frhs.2023.1159976)
Supplement: Supplementary file 1 [file Datasheet1.zip › Supplementary File 1.pdf]

Supplementary file 1: COREQ checklist

1 Consolidated criteria for reporting qualitative studies (COREQ): 32-item checklist (1)

No. Item	Guide questions/description	Reported on Page #
**Domain 1: Research team and reflexivity**		
Personal Characteristics		
1. Interviewer/facilitator	Which author/s conducted the interview or focus group?	Methods, page 3; Supplementary file 2
2. Credentials	What were the researcher's credentials? E.g. PhD, MD	Supplementary file 2
3. Occupation	What was their occupation at the time of the study?	Supplementary file 2
4. Gender	Was the researcher male or female?	Supplementary file 2
5. Experience and training	What experience or training did the researcher have?	Supplementary file 2
Relationship with participants		
6. Relationship established	Was a relationship established prior to study commencement?	Methods, page 3-4; Supplementary file 2
7. Participant knowledge of the interviewer	What did the participants know about the researcher? e.g. personal goals, reasons for doing the research	Methods, page 3; Supplementary file 2
8. Interviewer characteristics	What characteristics were reported about the inter viewer/facilitator? e.g. Bias, assumptions, reasons and interests in the research topic	Supplementary file 2
**Domain 2: study design**		
Theoretical framework		
9. Methodological orientation and Theory	What methodological orientation was stated to underpin the study? e.g. grounded theory, discourse analysis, ethnography, phenomenology, content analysis	Methods, page 4

No. Item	Guide questions/description	Reported on Page #
Participant selection		
10. Sampling	How were participants selected? e.g. purposive, convenience, consecutive, snowball	Methods, page 3
11. Method of approach	How were participants approached? e.g. face-to-face, telephone, mail, email	Methods, page 3
12. Sample size	How many participants were in the study?	Results, page 4
13. Non-participation	How many people refused to participate or dropped out? Reasons?	N/A
Setting		
14. Setting of data collection	Where was the data collected? e.g. home, clinic, workplace	Methods, page 3
15. Presence of non-participants	Was anyone else present besides the participants and researchers?	N/A
16. Description of sample	What are the important characteristics of the sample? e.g. demographic data, date	Results, page 4
Data collection		
17. Interview guide	Were questions, prompts, guides provided by the authors? Was it pilot tested?	Methods, page 3; Supplementary file 3
18. Repeat interviews	Were repeat interviews carried out? If yes, how many?	N/A
19. Audio/visual recording	Did the research use audio or visual recording to collect the data?	Methods, page 3-4
20. Field notes	Were field notes made during and/or after the interview or focus group?	N/A
21. Duration	What was the duration of the interviews or focus group?	Methods, page 3-4
22. Data saturation	Was data saturation discussed?	N/A
23. Transcripts returned	Were transcripts returned to participants for comment and/or correction?	N/A

No. Item	Guide questions/description	Reported on Page #
**Domain 3: analysis and findings**		
Data analysis		
24. Number of data coders	How many data coders coded the data?	Methods, page 4
25. Description of the coding tree	Did authors provide a description of the coding tree?	Supplementary file 5
26. Derivation of themes	Were themes identified in advance or derived from the data?	Methods, page 4
27. Software	What software, if applicable, was used to manage the data?	Methods, page 4
28. Participant checking	Did participants provide feedback on the findings?	Methods, page 4
Reporting		
29. Quotations presented	Were participant quotations presented to illustrate the themes/findings? Was each quotation identified? e.g. participant number	Results, pages 6-8; Supplementary file 4
30. Data and findings consistent	Was there consistency between the data presented and the findings?	Results, pages 4-6
31. Clarity of major themes	Were major themes clearly presented in the findings?	Results, pages 4-6
32. Clarity of minor themes	Is there a description of diverse cases or discussion of minor themes?	Discussion, pages 4-8

1. Tong A, Sainsbury P, Craig J. Consolidated criteria for reporting qualitative research (COREQ): a 32-item checklist for interviews and focus groups. International journal for quality in health care. 2007;19(6):349-57.
