# Supplementary material for: What contributes to the long-term implementation of an evidence-based early childhood intervention: a qualitative study from Germany
Source: Front Health Serv. 2024 Jan 19;3:1159976. doi: 10.3389/frhs.2023.1159976 (PMC10834770; doi:10.3389/frhs.2023.1159976)
Supplement: Supplementary file 1 [file Datasheet1.zip › Supplementary File 2.pdf]

Supplementary file 2: Research team and reflexivity

Characteristic	Description
Credentials	TJ, SK and MS are professors. TB holds a doctoral degree. MLS is a doctoral candidate. CS is a research associate.
Occupation	TB and MLS work at the Leibniz Institute for Prevention Research and Epidemiology -BIPS: TB as a senior researcher and MLS as a doctoral student and research assistant.<br>TJ works at the Carl von Ossietzky University of Oldenburg as a professor of Language and Communication and its Special Educational Promotion with Special Regard to Inclusive Educational Processes.<br>CS and SK work at the Ernst Abbe University of Applied Sciences in Jena: SK as a professor of social sciences, CS as a research associate. MS is employed at the Technical University Nuremberg as a professor for data science and empirical economics.
Gender	CS, MLS and TJ self-identified as women; MS, SK and TB self-identified as men.
Experience and training	The authors have a background in public health, health economics and educational psychology, and have worked with qualitative and quantitative data.
Relationship between participants and researchers	MLS, TJ, SK, CS and MS did not know any of the participants prior to the study. At study onset, MLS and TB presented the planned interview study to the Pro Kind staff during an annual network meeting run by Pro Kind (Bremen and Brunswick).
Participant knowledge of the interviewer	MLS is the only person who conducted the interviews. The participants knew that the interviewer was a researcher at the Leibniz Institute for Prevention Research and Epidemiology - BIPS. They were provided with written information about the aims of the project.
Interviewer characteristics	MLS is a psychologist (M.Sc.) and her research interest is child health, health inequalities and target group-specific adaptation and implementation of outreach prevention programs.
