# Supplementary material for: What contributes to the long-term implementation of an evidence-based early childhood intervention: a qualitative study from Germany
Source: Front Health Serv. 2024 Jan 19;3:1159976. doi: 10.3389/frhs.2023.1159976 (PMC10834770; doi:10.3389/frhs.2023.1159976)
Supplement: Supplementary file 1 [file Datasheet1.zip › Supplementary File 3.pdf]

Supplementary file 3: Interview guides (English translation on page 6)

1 Interview guide I (target group - program implementers): Original, German version

Einstieg: Zu Beginn möchte ich Sie bitten, kurz von Ihrer Tätigkeit bei Pro Kind zu erzählen und anschließend zu erläutern, was Sie im Kontext Ihrer Arbeit unter dem Begriff „Netzwerkarbeit" verstehen.

Entwicklung des Netzwerks:

- Wenn Sie an den Aufbau Ihres eigenen Netzwerks denken, gibt es da Bereiche, in denen sich die Zusammenarbeit mit verschiedenen Partner*innen oder Institutionen verändert hat?

- Fallen Ihnen Bereiche ein, in denen sich Ihr Netzwerk verkleinert oder vergrößert hat?
- Welche Faktoren führten Ihrer Meinung dazu, dass sich Ihr Netzwerk in den einzelnen Bereichen verändert hat/ oder nicht verändert hat?

Spezifische Nachfragen zur Entwicklung der Vernetzungsbereiche:

- Eine Herausforderung am Anfang des Pro Kind Programms ist es ja zunächst, die Familien bzw. Mütter zu erreichen und in das Programm mit einzubinden. Dabei spielt auch die Netzwerkarbeit eine bedeutende Rolle. Konnten Sie über die Zeit beobachten, dass sich im Hinblick auf die Erreichbarkeit der Mütter die Zusammenarbeit mit anderen Institutionen, sozialen Diensten oder professionellen Akteur*innen verändert hat?
- Ein Ziel des Programms Pro Kind ist es, die Eltern bei dem Wiedereinstieg in Ausbildung und Beruf zu unterstützen. Wie hat sich in diesem Punkt die Zusammenarbeit mit anderen Institutionen, sozialen Diensten, professionellen Akteur*innen entwickelt?
- Während der Begleitung der Familie geht es auch darum, die Kinder in die Krippe und Kita zu vermitteln. Wie hat sich in diesem Bereich Ihr Netzwerk über die Zeit entwickelt oder verändert?
- Während der Begleitung arbeiten Sie häufig auch mit zusätzlichen Unterstützungsangeboten zusammen, um die Familien individuell zu fördern. Hat sich Ihr Netzwerk im Hinblick auf diese zusätzlichen Unterstützungsangebote verändert?
- Am Ende der Begleitung werden Familien/ Mütter/ Kinder an weiterführende Unterstützungsangebote vermittelt. Wie hat sich in diesem Bereich Ihr Netzwerk über die Zeit entwickelt oder verändert?

Aktuelles Netzwerk: Nun möchte ich Sie gerne zu Ihren Partner*innen der Frühen Hilfen befragen, mit denen Sie als Pro Kind Mitarbeiter*in aktuell zusammenarbeiten.

- Welche Institutionen, sozialen Dienste oder professionellen Akteur*innen sind von besonderer Bedeutung für Ihren Arbeitsalltag und warum?

  - Wer zählt in Ihrem Arbeitsalltag zu den wichtigsten Partner*innen? Warum?
  - Worum geht es bei der Zusammenarbeit?
  - Wie häufig haben Sie Kontakt?

- In welcher Form tauschen Sie sich aus bzw. kommunizieren Sie miteinander?
- Würden Sie die Beziehung eher auf organisationaler Ebene oder persönlicher Ebene einordnen?
- Gibt es auch vertraglich geregelte Kooperationen in Ihrem Netzwerk?

Aufrechterhaltungsfrage: (falls der Person wenige Partner*innen einfallen)

- Gibt es Kooperationspartner*innen, mit denen Sie im Hinblick auf…
  1) die Vermittlung von Kinderbetreuungsmöglichkeiten
  2) die Aufnahme von Berufsausbildung & Erwerbstätigkeit
  3) Weitervermittlung am Ende der Betreuung … zusammenarbeiten?

- Gibt es Bereiche, in denen Ihre Kooperationsstrukturen besonders gut ausgebaut sind?
  - Was könnten Gründe dafür sein?
  - Wie wirkt sich das auf Ihren Arbeitsalltag und Pro Kind aus?

- Gibt es Bereiche, in denen es Ihnen an Kooperationspartner*innen mangelt?
  - Woran könnte das liegen?
  - Wie wirkt sich das auf Ihren Arbeitsalltag und Pro Kind aus?

Förderliche und hinderliche Faktoren für eine nachhaltige Wirksamkeit und Implementierung

- Welche Faktoren sind Ihrer Meinung nach ausschlaggebend und förderlich für eine erfolgreiche Zusammenarbeit mit Ihren Kooperationspartner*innen?

- Wie muss aus Ihrer Sicht mit bestehenden Initiativen, Netzwerkpartner*innen und Strukturen kommuniziert und kooperiert werden, damit sich eine bestmögliche Zusammenarbeit ergibt?
- Haben Sie vielleicht eine konkrete Situation oder einen konkreten Fall in Erinnerung, in dem die Zusammenarbeit mit anderen Akteur*innen besonders konstruktiv verlief/ verläuft? Was glauben Sie - woran lag/ liegt das?
- Welche Faktoren sind Ihrer Meinung nach hinderlich für den Netzwerkaufbau und eine erfolgreiche Zusammenarbeit mit Ihren Kooperationspartner*innen?
- Haben Sie vielleicht eine konkrete Situation oder einen konkreten Fall in Erinnerung, in dem es zu Unstimmigkeiten/ Brüchen mit anderen Kooperationspartner*innen kam? Wie wurde damit umgegangen und wurde der Konflikt gelöst?

Nun haben wir recht viel über Ihre Kooperationspartner*innen und das Netzwerk von Pro Kind gesprochen. Darüber hinaus gibt es sicherlich viele weitere Faktoren die Ihre Arbeit beeinflussen.

- Wenn Sie an Ihren Arbeitsalltag und die Betreuung der Familien denken, wo sehen Sie Entwicklungsbedarf von Pro Kind?

- Was ist aus Ihrer Sicht besonders wichtig, damit möglichst viele Familien erreicht und bestmöglich versorgt werden können?
- Wo hakt es im Berufsalltag und wie könnten diese Probleme gelöst werden?
- Haben Sie den Eindruck, dass die Familien am Ende der „Präventionskette“ von Pro Kind profitieren? Warum (nicht)?

Abschlussfragen:

- Fallen Ihnen noch Personen, Institutionen, Netzwerkpartner*innen ein, die wir zu dieser Thematik befragen sollten? Warum?

- Wir sind nun am Ende des Interviews angelangt. Gibt es von Ihnen noch etwas, das bisher im Interview noch nicht zur Sprache gekommen ist, was Ihnen aber wichtig ist?

2 Interview guide II (target group - stakeholders): Original, German version

Einstieg: Zu Beginn möchte ich Sie bitten, kurz von Ihrer aktuellen Tätigkeit im Kontext der Frühen Hilfen zu berichten.

- Was sind Ihre bisherigen Berührungspunkte mit Pro Kind?
  - Woher kennen Sie Pro Kind?
  - Was haben Sie bisher mit Pro Kind zu tun gehabt?

Entwicklung des Netzwerks: Sie kennen das Programm Pro Kind schon länger und sind beruflich in dem Feld der Frühen Hilfen tätig.

- Was meinen Sie – welchen Stellenwert hat Pro Kind im Bereich der Frühen Hilfen in Bremen/ Braunschweig?
  - Was trägt Pro Kind zur Versorgung im Bereich der Frühen Hilfen bei?
  - Worin liegt die Besonderheit des Programms?
  - Welche Impulse, Ideen oder Konzepte hat Pro Kind in die Landschaft der Frühen Hilfen mit eingebracht?

- Wie würden Sie die Zusammenarbeit mit Pro Kind beschreiben?
  - Falls der/ die Befragte nicht mit Pro Kind zusammenarbeitet: Wie würden Sie die Zusammenarbeit von Pro Kind mit anderen Institutionen oder Akteur*innen beschreiben?
  - Würden Sie sagen, dass sich die Zusammenarbeit über die Zeit verändert hat? (Wenn ja - in welchen Bereichen? Warum?)
  - Gibt es Ihrer Meinung nach Kooperationspartner*innen, die von besonderer Bedeutung für Pro Kind sind? Warum?
  - Gibt es Ihrer Meinung nach „Lücken“ im Netzwerk von Pro Kind oder mangelt es in bestimmten Bereichen an Kooperationspartner*innen? Woran könnte das liegen?

- In Bremen/ Braunschweig gibt es ja auch Angebote im Bereich der Frühen Hilfen, die dem Pro Kind Programm inhaltlich sehr ähnlich sind. Wie würden Sie die Beziehung zu diesen Angeboten beschreiben?
  - Können Sie die Beziehung zu diesen Angeboten einschätzen?
  - Arbeiten die Angebote zusammen oder gibt/ gab es eher eine Art „Konkurrenzsituation“?
  - Hat sich das Verhältnis über die Zeit verändert?

Förderliche und hinderliche Faktoren für eine nachhaltige Wirksamkeit und Implementierung

- Wie effektiv greifen Ihrer Meinung nach die Räder des Netzwerks von Pro Kind ineinander, damit die Familien bestmöglich versorgt werden?
  - Wo hakt es gegebenenfalls?

- Gibt es neben der effektiven Netzwerkarbeit weitere Aspekte, die Ihrer Meinung nach von besonderer Bedeutung für eine nachhaltige Wirksamkeit von Pro Kind sind?

- Haben Sie den Eindruck, dass die Familien am Ende der „Präventionskette“ von Pro Kind profitieren? Warum (nicht)?
    - Was ist aus Ihrer Sicht besonders wichtig, damit möglichst viele Familien erreicht und bestmöglich versorgt werden können?
    - In welchen Bereichen gibt es ggf. Entwicklungspotential?

Abschlussfragen:

- Fallen Ihnen noch Personen, Institutionen, Netzwerkpartner*innen ein, die wir zu dieser Thematik befragen sollten? Warum?

- Wir sind nun am Ende des Interviews angelangt. Gibt es von Ihnen noch etwas, das bisher im Interview noch nicht zur Sprache gekommen ist, was Ihnen aber wichtig ist?

3 Interview guide I (target group - program implementers): English translation

Introduction: At the beginning, I would like to ask you to tell us briefly about your work at Pro Kind and then to explain what you understand by the term "networking" in the context of your work.

Network development:

- When you think about the development of your own network, are there areas where the collaboration with different partners or institutions has changed over time?
    - Can you think of areas where your network has decreased or increased?
    - In your opinion, what factors led to your network changing/ not changing in each area?

    Specific follow-up questions about the development of networking areas:

    - One challenge at the beginning of the Pro Kind program is, first of all, to reach families or mothers and involve them in the program. Networking also plays an important role in this. Have you noticed over time that cooperation with other institutions, social services or professional actors has changed with regard to the reach of the mothers?
    - One of the aims of the Pro Kind program is to support parents in getting back into training and work. How has cooperation with other institutions, social services and professional actors developed in this respect?
    - While supporting the family, it is also about placing the children in the daycare center. How has your network developed or changed over time in this area?
    - While supporting the family, you also often work with additional support services to provide individualized support to families. Has your network changed with regard to these additional support services?
    - At the end of the home visiting program, families/mothers/children are referred to additional support services. How has your network developed or changed over time in this area?

Current network: Now I would like to ask you about your partners in the field of early childhood interventions with whom you currently work as a Pro Kind employee.

- Which institutions, social services or professional actors are of particular importance for your everyday work and why?
    - Who are the most important partners in your daily work? Why?
    - What is the collaboration about?
    - How often do you have contact?
    - In what form do you engage or communicate with each other?
    - Would you classify the relationship rather on an organizational level or on a personal level?
    - Are there also contractually regulated collaborations in your network?

Specific follow-up questions (if the person can think only of few partners)

- Are there any collaborative partners with whom you work with regard to...
  1) arranging child care
  2) starting vocational training & employment
  3) referral at the end of the home visiting program

- Are there areas where your collaborative structures are particularly well developed?
  - What could be the reasons for this?
  - How does this affect your everyday work and the Pro Kind program?

- Are there areas in which you lack cooperation partners?
  - What could be the reasons for this?
  - How does this affect your everyday work and the Pro Kind program?

Facilitators and barriers for sustainable effectiveness and implementation:

- In your opinion, what factors are decisive and conducive to a successful collaboration with your cooperation partners?
  - How do you think it is necessary to communicate and cooperate with existing initiatives, network partners and structures in order to achieve the best possible cooperation?
  - Do you have a concrete situation or case in mind in which the cooperation with other partners was particularly constructive? What do you think was/is the reason for this?
  - In your opinion, which factors hinder the establishment of a network and successful cooperation with your cooperation partners?
  - Do you have a concrete situation or case in mind in which there were disagreements with other cooperation partners? How was this handled and was the conflict resolved?

Now we have talked quite a bit about your cooperation partners and the Pro Kind network. Beyond that, there are certainly many other factors that influence your work.

- When you think about your everyday work and the care of the families, where do you see a need for development of the Pro Kind program?
  - What do you think is particularly important so that as many families as possible can be reached and cared for in the best possible way?
  - Where do problems arise in your everyday work and how could these problems be solved?
  - Do you have the impression that the families benefit from Pro Kind? Why (not)?

Closing questions:

- Can you think of any other persons, institutions, network partners that we should ask about this topic? Why?
- We have now reached the end of the interview. Is there anything else that hasn't been mentioned in the interview, but which is important to you?

4 Interview guide II (target group - stakeholders): English Translation

Introduction: At the beginning, I would like to ask you to briefly report on your current professional activities in the context of early childhood interventions.

- What are your previous points of contact with Pro Kind?
  - How do you know Pro Kind?
  - What have you had to do with Pro Kind so far?

Network development: You have known the Pro Kind program for some time and are professionally active in the field of early childhood interventions.

- What do you think - what is the significance of program Pro Kind in the field of early childhood interventions in Bremen/Brunswick?
  - What does Pro Kind contribute to the services in the field of early childhood interventions?
  - What is special about the program?
  - What impulses, ideas or concepts has Pro Kind contributed to the field of early childhood interventions?

- How would you describe the cooperation with Pro Kind?
  - If the interviewee does not work with Pro Kind: How would you describe Pro Kind's cooperation with other institutions or actors?
  - Would you say that the cooperation has changed over time? (If yes - in which areas? Why?)
  - In your opinion, are there cooperation partners who are of particular importance for Pro Kind? Why?
  - In your opinion, are there "gaps" in the Pro Kind network or is there a lack of cooperation partners in certain areas? What could be the reason for this?

- In Bremen/Brunswick, there are also programs in the field of early childhood interventions that are very similar to the Pro Kind program. How would you describe the relationship with these services?
  - Can you assess the relationship with these services?
  - Do the programs work together or is there/was there rather a kind of "competitive situation"?
  - Has the relationship changed over time?

Facilitators and barriers for sustainable effectiveness and implementation:

- In your opinion, how effectively is the Pro Kind network developed to provide the best possible care for families?
    - Where do problems occur, if any?
- Apart from effective networking, are there other aspects that you think are of particular importance for the sustainable effectiveness of Pro Kind?
- Do you have the impression that the families benefit finally from Pro Kind? Why (not)?
    - In your opinion, what is particularly important to ensure that as many families as possible are reached and provided with the best possible care?
    - In which areas, if any, is there potential for development?

Closing questions:

- Can you think of any other persons, institutions, network partners that we should ask about this topic? Why?

- We have now reached the end of the interview. Is there anything else that hasn't been mentioned in the interview, but which is important to you?
