# Supplementary material for: What contributes to the long-term implementation of an evidence-based early childhood intervention: a qualitative study from Germany
Source: Front Health Serv. 2024 Jan 19;3:1159976. doi: 10.3389/frhs.2023.1159976 (PMC10834770; doi:10.3389/frhs.2023.1159976)
Supplement: Supplementary file 1 [file Datasheet1.zip › Supplementary File 4.pdf]

frontiers

Supplementary file 4: Translations of quotations used in the manuscript

Domain	German (Original raw data)	English translation
**Intervention Characteristics**	Gerade auch Familien, die zuerst mit Gegenwehr irgendwie waren, weil sie halt das Programm zuerst nicht so richtig einschätzen konnten, dass die nachher gesagt haben, dass das richtig gut war.	Especially families, who at first were somehow resistant, because they could not really grasp the program at first, said afterwards that it was really good.
	… aber die haben ja schon eine klar umrissene Zielgruppe. Und viele meiner Klienten, zum Beispiel, passen da gar nicht rein. Also es sind nicht immer Erstgebärende, die diese Unterstützung bräuchten. Es sind gerade oft auch Zweit- und Drittgebärende (…).	… but they already have a clearly defined target group. And many of my clients, for example, don't fit in at all. So it's not always first-time mothers who need this support. It is often second and third-time mothers (…).
**Inner Setting**	der Impuls der fachlichen Begleitung und (…) der relativ förderlichen Arbeitsbedingungen der Fachkräfte, ja? Also Fachberatung, regelmäßig Fortbildungen, regelmäßig feste Anstellungen der Kolleginnen im Gegensatz zu den Familienhebammen bei dem, ah nein, die sind auch angestellt, aber in allen anderen Bundesländern sind ja Familienhebammen nicht angestellt, sondern arbeiten honorarmäßig, was eine Katastrophe ist für diese Arbeit..	… the impulse of the professional support and (…) the relatively conducive working conditions of the professionals, right? So professional advice, regular training, regular permanent employment of colleagues in contrast to the family midwives (...) in all other states, family midwives are not employed, but work on a fee basis, which is a disaster for this work.
	Und Pro Kind ist ja tatsächlich auch aktiv ja auch so an kleineren Projekten. Also hatte ich ja auch schon erlebt, dass die Leitung mit dabei saß… also bei irgendwelchen Flyergestaltungen zur einfachen Sprache dann, oder ich meine, dass sie auch dabei waren, als diese Karten für Smartphonenutzung und Kinderbetreuung irgendwie entwickelt worden sind, dass die da auch mit bei saßen und sich aktiv eingebracht haben.	And Pro Kind is actually also active in smaller projects. So, I have already experienced that the management participated in the designing of the flyer in simple language, or I think that they were also there when these cards for smartphone use and childcare were somehow developed, that they were also present and actively contributed.
	Also ich erlebe Pro Kind als einen der großen Mitspieler. Also da denke ich sofort dann, okay, große Organisation, viele Kollegen, großer Bekanntheitsgrad auch, und sehr etabliert, in meiner Wortwahl.	I experience Pro Kind as one of the big players. So, then I immediately think, okay, big organization, many colleagues, widely known too, and very established, in my choice of words.

Domain	German (Original raw data)	English translation
**Outer Setting**	… was mir immer wieder begegnet, (…) ist das Sprachliche, die Sprachbarriere. Also in vielen Familien ist halt die Muttersprache präsent, es wird wenig Deutsch gekonnt. Und natürlich haben die Hebammen auch nicht alle diese Sprachkenntnisse. Und da glaube ich auch noch mal, bei den Mitarbeiterinnen zu gucken, können wir doch auch noch mal Personen akquirieren, die gegebenenfalls auch die eine oder andere Sprache sprechen. (…). Ich glaube, das würde Pro Kind sich wahrscheinlich auch selber wünschen.	… what I experience again and again (...) is the language, the language barrier. So, in many families, the mother tongue is present, there is little German proficiency. And of course, not all midwives have these language skills. And I think we need to look again at the employees, can we also hire people who speak one language or another. (...). I think that would probably also be Pro Kind`s wish.
	Also Netzwerkarbeit ist ganz wichtig. Pro Kind ohne Netzwerkarbeit würde überhaupt nicht funktionieren (…). Der Zugang gelingt nur über unsere Kooperationspartner. Und dann gibt es ja spezifische Themen. Das heißt wir verstehen uns für spezifische Themen als Lotsinnen. Dass wir den Familien eben sagen können, für das Problem könnt ihr dahin gehen (…) und dass wir gemeinsam gucken, dass die Familien es schaffen (…) dann passgenaue Hilfe und Unterstützung auch zu bekommen.	So, networking is very important. Pro Kind without networking wouldn't work at all (...). Access is only possible through our stakeholders. And then there are specific issues. That means we see ourselves as guides for specific issues. Meaning we can tell the families that they can go there for the problem (...) and that we work together to ensure that the families manage to receive the help and support they need.
	… also dadurch, dass es nicht nur in einzelnen Ortsteilen als Programm präsent ist, sondern in ganz Bremen, ist es bekannt und damit auch im kommunalen Netzwerk ein etablierter Partner.	… by being present as a program not only in individual districts, but throughout Bremen, it is well known and thus also an established partner in the municipal network.
	Es ist schwieriger die Frauen für das Projekt zu begeistern. Weil sage ich jetzt Ihnen, in den Augen der jungen Mütter, die eventuell schon Erfahrungen mit dem Jugendamt als Kind gemacht, ist die Ähnlichkeit mit der ambulanten Jugendhilfe zu hoch.	It is more difficult to motivate women to join the project. Because I tell you now, in the eyes of the young mothers, who may have already had experiences with the youth and welfare office as a child, the similarity with outpatient child protection service is too large.
	Wir müssen jedes Jahr neu beantragen, neu gucken und das nimmt ganz viel Energie auch.	We have to re-apply every year, check again and that takes a lot of energy as well.
