# Supplementary material for: What contributes to the long-term implementation of an evidence-based early childhood intervention: a qualitative study from Germany
Source: Front Health Serv. 2024 Jan 19;3:1159976. doi: 10.3389/frhs.2023.1159976 (PMC10834770; doi:10.3389/frhs.2023.1159976)
Supplement: Supplementary file 1 [file Datasheet1.zip › Supplementary File 5.pdf]

Supplementary file 5: Coding frame example

**CFIR-Domain**	**Construct/theme**	**Description**	**Codes**	**Examples of quotes**<br>(referring to codes in bold)
Intervention Characteristics	Evidence strength & quality	Mitarbeitende und Stakeholder halten die Intervention für wirksam. Aus Ihrer Perspektive profitieren die Familien von Pro Kind (anhand von Beobachtungen, Rückmeldungen etc.)	**#Wirksamkeit Perspektive Stakeholder**	*Gerade auch Familien, die zuerst mit Gegenwehr irgendwie waren, weil sie halt das Programm zuerst nicht so richtig einschätzen konnten, dass die nachher gesagt haben, dass das richtig gut war.*
	Relative advantage	Ansatz des Programms wird als Alleinstellungsmerkmal und Vorteil gegenüber anderen Institutionen genannt: Erstgebärende, früher Einstieg und Dauer des Programms, Ganzheitlichkeit, Freiwilligkeit	#Besonderheit Programmdauer<br><br>#Besonderheit Erstgebärende<br><br>**#Besonderheit früher Programmeinstieg**<br><br>#Eltern sind die Expert*innen<br><br># Eltern sind die Auftraggeber*innen	*Was bei Pro Kind ja wirklich eigentlich toll ist, ist dieser Zeitpunkt, dass man wirklich früh genug anfangen kann, mit den Familien zu arbeiten, wo sie noch bereit sind, auch etwas zu verändern. Das ist schon richtig so, glaube ich, und auch stark von dem Programm… also was es wirklich gut kann.*
